# Chemotherapy for Locally Advanced Pancreatic Cancer: The Dilemma of David Versus Goliath After NEOPAN

**DOI:** 10.1200/JCO-25-01517

**Published:** 2025-12-04

**Authors:** Federico Nichetti, Letizia Procaccio, Sara Lonardi, Francesca Bergamo

**Affiliations:** Federico Nichetti, MD; Letizia Procaccio, MD, PhD; Sara Lonardi, MD; and Francesca Bergamo, MD, Istituto Oncologico Veneto IOV—IRCCS, Padova, Italy

## To the Editor:

The challenging PRODIGE 29-UCGI 26 (NEOPAN) trial^1^ was conducted in a population where randomized evidence is critically needed. NEOPAN represents a cornerstone for both future research and clinical practice in the management of locally advanced pancreatic cancer (LAPC). Some aspects merit further discussion to best interpret its results and guide the ongoing update of international guidelines.

NEOPAN was designed to detect a 3-month improvement in median progression-free survival (mPFS), from 5 to 8 months. This was a reasonable target, particularly in light of the phase III CONKO-007 trial interim analysis, which also reported an 8-month mPFS in the chemotherapy-alone arm, approximately 84% of whom received fluorouracil, oxaliplatin, and irinotecan (FOLFIRINOX).^2^ However, with the benefit of hindsight, the observed advantage was statistically significant but did not clearly cross the efficacy boundary: the trial arms slightly overperformed, especially for gemcitabine, as the observed mPFS gain was from 7.7 to 9.7 months in the experimental arm. Moreover, this PFS benefit did not translate into an overall survival (OS) advantage, which may prompt discussion regarding the clinical impact of the findings. It is important to note that the study was not powered to detect differences in this outcome and many factors may explain the lack of OS benefit.

Among patients receiving postprogression chemotherapy, 56% of those deemed fit for modified FOLFIRINOX (mFOLFIRINOX) but randomly assigned to gemcitabine were treated with second-line, multiagent, non–gemcitabine-based regimens (mFOLFIRINOX, FOLFOX, or FOLFIRI), whereas 44% of those in the mFOLFIRINOX arm received gemcitabine monotherapy at progression. In this regard, an analysis of PFS1 + PFS2 or of PFSratio,^3^ reflecting the impact of subsequent therapies, would be informative.

Importantly, NEOPAN reported equally low resection rates, accurately mirroring real-world practice. Given that long-term survival in LAPC is primarily driven by surgical resection,^2,4,5^ when this is not achievable, the therapeutic goal should shift toward palliation with the least possible harm.

In this context, treatment toxicity is crucial. NEOPAN provides reassuring evidence on the tolerability of mFOLFIRINOX when administered in experienced centers. However, an acknowledged limitation is the lack of information regarding granulocyte colony-stimulating factor (G-CSF) use. Given that grade ≥3 neutropenia was more frequent in the gemcitabine monotherapy arm, it is reasonable to infer that prophylactic G-CSF was used routinely with mFOLFIRINOX. This consideration is relevant when compared with once weekly regimens, which are often subject to treatment delays because of neutropenia, potentially affecting their efficacy. Another key missing data point concerns neurotoxicity, which often limits oxaliplatin use and likely contributed to its lower dose intensity in NEOPAN. Recent evidence suggests that maintaining at least 80% relative dose intensity may be important for efficacy of triplet regimens.^6^ Therefore, stating that mFOLFIRINOX was better tolerated than gemcitabine monotherapy, without reporting these key toxicity data, may be misleading.

Age stratification is another key consideration. Although the trial enrolled patients age up to 84 years—rarely candidates for triplet regimens in routine practice—the use of a 60-year cutoff is difficult to interpret, particularly given the seemingly greater benefit of mFOLFIRINOX in older patients. Analyses with age as a continuous variable and in narrower bands (eg, 5-year intervals) would offer clearer insight. Notably, ongoing trials in LAPC adopting mFOLFIRINOX (eg, ClinicalTrials.gov identifier: NCT06897644) require TRIBE^7^-like criteria, with an upper age cutoff of 75 years and an Eastern Cooperative Oncology Group performance status of 0 for patients older than 70 years.

Overall, while it is plausible that mFOLFIRINOX could provide an OS benefit over gemcitabine alone in selected patients, a trial powered to robustly detect this would require an impractically large sample size.

What could then the next steps be? First, the potential benefit of even more intensive strategies should be explored. The quadruplet PAXG regimen showed encouraging results in the PACT-21 trial, directly compared with mFOLFIRINOX in resectable and borderline resectable pancreatic ductal adenocarcinoma.^8^ Second, molecular stratification is essential to move toward a right treatment for the right patient approach. Beyond germline *BRCA1/2* mutations, which mandate the use of platinum-based regimens,^9^ recent work has led to the development of transcriptomic signatures capable of accurately predicting sensitivity to FOLFIRINOX or gemcitabine.^10^ These signatures have been validated in the adjuvant setting, and their application in LAPC warrants further investigation. Finally, novel agents, including *RAS* inhibitors,^11^ are on the horizon and could shift the treatment paradigm. Should they prove to be effective, evaluating their role in LAPC will be a natural next step.

Until these developments mature, and while awaiting the further details from NEOPAN, we have suggested that these nuances highlight the importance of clinical judgment: in fit patients requiring rapid disease control, the choice of mFOLFIRINOX is appropriate; for frailer cases with no chance of surgical exploration, gemcitabine monotherapy remains a reasonable choice.
